# Treatment and re-treatment results of HCV patients in the DAA era

**DOI:** 10.1371/journal.pone.0232773

**Published:** 2020-05-05

**Authors:** Felix Piecha, Jan-Michael Gänßler, Ann-Kathrin Ozga, Malte H. Wehmeyer, Julia Dietz, Johannes Kluwe, Alena Laschtowitz, Johann von Felden, Martina Sterneck, Sabine Jordan, Sven Pischke, Ansgar W. Lohse, Julian Schulze zur Wiesch

**Affiliations:** 1 I. Department of Medicine, University Medical Center Hamburg-Eppendorf, Hamburg, Germany; 2 German Center for Infection Research (DZIF), Partner Site Hamburg-Lübeck-Borstel-Riems, Hamburg, Germany; 3 Center for Experimental Medicine, Institute of Medical Biometry and Epidemiology, University Medical Center Hamburg-Eppendorf, Hamburg, Germany; 4 Department of Internal Medicine 1, University Hospital Frankfurt, Frankfurt, Germany; 5 German Center for Infection Research (DZIF), External Partner Site Frankfurt, Frankfurt, Germany; University of Cincinnati College of Medicine, UNITED STATES

## Abstract

**Background:**

Re-treatment in patients with a chronic hepatitis C virus (HCV) infection and a previous failure to direct-acting antiviral (DAA) treatment remains a challenge. Therefore, we investigated the success rate of treatment and re-treatment regimens used at our center from October 2011 to March 2018.

**Methods:**

A retrospective analysis of DAA-based HCV therapies of 1096 patients was conducted. Factors associated with a virological relapse were identified by univariable and multivariable logistic regression, treatment success of the re-treatment regimens was evaluated by an analysis of sustained virological response (SVR) rates in patients with a documented follow-up 12 weeks after the end of treatment.

**Results:**

Of 1096 patients treated with DAA-based regimens, 91 patients (8%) were lost to follow-up, 892 of the remaining 1005 patients (89%) achieved an SVR12. Most patients (65/113, 58%) who experienced a virological relapse received an interferon-based DAA regimen. SVR rates were comparable in special cohorts like liver transplant recipients (53/61, 87%) and people with a human immunodeficiency virus (HIV) coinfection (41/45, 91%). On multivariable analysis, interferon-based DAA therapy was associated with treatment failure (odds ratio 0.111, 95%-confidence interval 0.054–0.218) among others. One hundred seventeen patients with multiple DAA treatment courses were identified, of which 97 patients (83%) experienced a single relapse, but further relapses after two (18/117, 15%) or even three (2/117, 2%) treatment courses were also observed. Eighty-two of 96 (85%) re-treatment attempts with all-oral DAA regimens were successful after an initial treatment failure.

**Conclusion:**

Overall, DAA re-treatments were highly effective in this real-world cohort and only a minority of patients failed more than two treatment courses. Switching to–or addition of–a new drug class seem to be valid options for the re-treatment of patients especially after failure of an interferon-based regimen.

## Introduction

Since the introduction of direct-acting antivirals (DAA) for the treatment of chronic hepatitis C virus (HCV) infection, sustained virological response (SVR) rates have steadily and incrementally increased, now reaching >90% even in formerly difficult to treat populations [1–4]. However, due to the implementation of broad HCV eradication programs worldwide, considerable numbers of patients who have failed an initial DAA therapy are to be expected, and data on re-treatment strategies are still scarce [5–8]. So far, most studied re-treatment attempts were carried out by combining sofosbuvir with a different DAA class than the patient had formerly received, an extension of treatment duration or the addition of ribavirin [9].

Recently, an effective single-pill re-treatment regimen (sofosbuvir/velpatasvir/voxilaprevir) for DAA-experienced patients has been approved by US and European regulatory authorities [10], but accessibility and remaining high costs limit its universal use especially in lower-income countries. Additionally, the combination regimen glecaprevir/pibrentasvir has also been approved in some regions for selected patients in whom a previous interferon-free DAA treatment course had failed [11]. However, as it contains a protease inhibitor, its use is contraindicated in patients with decompensated liver cirrhosis.

This descriptive analysis of all DAA-based HCV therapies carried out at our center gives an overview of the real-world success rate of every DAA-based regimen that has been introduced since 2011, including in special cohorts like patients with liver cirrhosis, people with human immunodeficiency virus (HIV) and liver-transplanted patients. Furthermore, we assessed factors that were associated with DAA treatment failure and describe the results of re-treatment regimens used in patients in whom a prior DAA-based treatment course had failed.

## Materials and methods

### Patients

The study was approved by the local ethics committee (Ethikkommission Ärztekammer Hamburg, reference WF-015/18) in accordance with the principles of the declaration of Helsinki.

All patients who received a DAA-based antiviral treatment at the University Medical Center Hamburg-Eppendorf between October 2011 and March 2018 were identified by database search. The University Medical Center Hamburg-Eppendorf is a large medical center in northern Germany which covers a catchment area of more than 5 million inhabitants. As a tertiary referral center, it provides full service in all fields of medicine, including liver transplantation. At our outpatient clinic for viral hepatitis, we annually see approximately 1000 HCV patients, providing full service with regard to counseling, testing, treatment and surveillance.

For all patients, information on demographics, presence of liver cirrhosis, hepatocellular carcinoma (HCC), HIV coinfection, previous liver transplantation, interferon (IFN) treatment experience, HCV genotype (GT) and the type and duration of the treatment regimen used, was collected. GT testing was carried out in all patients prior to treatment using real-time PCR sequencing of the 5-UTR and NS5B region using an in-house protocol, and additionally the protease +/- NS5B region in unclear cases. SVR was defined as a documented negative HCV-PCR 12 weeks after the end of treatment (EoT). A virological relapse was defined as the detection of viral replication 12 weeks after EoT. Patients starting a therapy but without another serological test of viral RNA at least 12 weeks after EoT were defined as lost to follow-up (LTFU). In patients receiving multiple DAA courses, each treatment course was analyzed individually so that the data displayed in this paper are number of therapies. Discrepancies in numbers of patients with a virological relapse and patients receiving re-treatment at our center are explained by patients receiving a first DAA therapy at a different center or as part of a protease inhibitor-containing clinical trial regimen (telaprevir and ritonavir). The choice of a treatment regimen was made by the individual provider in consideration of drug availability at a certain timepoint, expected results and cost-effectiveness after consultation with an internal hepatitis board.

Cirrhosis was diagnosed by biopsy or non-invasively using transient elastography (TE, Fibroscan, Echosens, Paris, France) with a liver stiffness (LS) cut-off value of >12.5 kPa at baseline in accordance with the literature [12, 13] or clinical signs of portal hypertension. A baseline LS measurement was available in 864 patients.

### Amplification and sequencing analysis

Resistance-associated substitution (RAS) testing was performed as part of the clinical routine at the University Hospital Frankfurt prior to the initiation of or after failure to a previous DAA treatment as previously described [14]. Therefore, RAS testing was only available in a pre-selected, non-representative subgroup of patients who were *a priori* considered to be difficult to treat, e.g. due to their specific GT, presence of liver cirrhosis or prior DAA treatment failure. For RAS analysis, the HCV NS3, NS5A and NS5B DAA target regions were amplified using nested PCR. PCR products were population-based sequenced on an ABI Prism 3130xl Genetic Analyzer (Applied Biosystems, Foster City, CA). Details on primers, PCR conditions, sequencing as well as on the analysis of relevant RASs have been published previously [14, 15]. The sensitivity of population-based sequencing is approximately 15% for minority HCV variants in the quasispecies [16]. The real-time PCR has an approximate sensitivity of 1000 IU/mL.

### Statistical analysis

Categorical data are displayed as percentages and counts, mean and median values with the corresponding standard deviation (SD) or interquartile range (IQR) were calculated for continuous data using Excel 2016 (Microsoft Inc., Redmond, WA) and SPSS Statistics Version 25 (IBM Corp., Armonk, NY). SVR rates were calculated by dividing the number of patients with a successful therapy by all patients with a documented follow-up at least 12 weeks after EoT. Factors related to a virological relapse were identified by univariable logistic regression, followed by a multivariable logistic regression that included all parameters with a p-value <0.1 in the univariable regression. Given the exploratory character of these analyses, no adjustment for multiple testing was conducted and the significance level was set to be 0.05 for all calculations. Figure design and statistical testing were carried out using SPSS Version 25, R Version 3.5.1 (R Core Team, 2018), and GraphPad Prism Version 8.0 (GraphPad Software, La Jolla, CA).

## Results

### Patient characteristics

From 2011 to 2018, 1096 patients were treated with DAA-based therapies at our center, of which 218/1096 (20%) were IFN-based and 878/1096 (80%) IFN-free regimens (Table 1). Patients were predominantly male (628/1096, 57%) with a mean age of 52.3 ± 12.7 years. 384 (35%) patients were IFN-experienced, and 320 patients (29%) had liver cirrhosis. Fewer patients were liver transplant recipients (63/1096, 6%) or people with HIV (49/1096, 4%). GT1 was most common (791/1096, 72%), followed by GT3 (173/1096, 16%) and GT4 (71/1096, 6%). In the entire cohort, an SVR was achieved in 892/1005 (89%) of cases, with a higher proportion of SVR in patients receiving IFN-free regimens compared to patients receiving IFN-based therapies (752/800, 94% SVR vs. 140/205, 68% SVR, respectively). In 91/1096 (8%) of cases, patients were LTFU, including nine patients who died during therapy.

**Table 1:** Baseline characteristics of patients treated for chronic HCV infection with DAA-based regimens between October 2011 and March 2018.

	Complete cohort n = 1096 (%)	IFN-based DAA regimens n = 218 (%)	IFN-free DAA regimens n = 878 (%)
Age (y, mean ± SD)	52.3 ± 12.7	49.3 ± 11.8	53.1 ± 12.9
Sex (male/female)	628 (57) / 468 (43)	142 (65) / 76 (35)	486 (55) / 392 (45)
Liver transplanted patients	63 (6)	9 (4)	54 (6)
People with HIV	49 (4)	11 (5)	38 (4)
Presence of HCC	24 (2)	4 (2)	20 (2)
Liver cirrhosis	320 (29)	66 (30)	254 (29)
Child-Pugh score A/B/C	238/79/3	58/8/0	180/71/3
Median MELD score (IQR)	8.0 (7.0, 10.5)	7.5 (7.0, 9.0)	8.0 (7.0, 11.0)
IFN-experienced	384 (35)	117 (54)	267 (30)
HCV Genotype			
1 (a/b/c/unclassified)	791 [72] (342/418/5/26)	175 [80] (73/91/2/9)	616 [70] (270/327/3/16)
2	44 (4)	0 (0)	44 (5)
3	173 (16)	25 (11)	148 (17)
4	71 (6)	13 (6)	58 (7)
5	3 (0)	1 (0)	2 (0)
6	6 (1)	3 (1)	3 (0)
1/3 coinfection	2 (0)	0 (0)	2 (0)
2k/1b	2 (0)	0 (0)	2 (0)
Unknown	4 (0)	1 (0)	3 (0)
**Outcome parameters**			
Lost to follow-up	91 (8)	13 (6)	78 (9)
Documented 12-week FU after EoT	1005 (92)	205 (94)	800 (91)
Virological relapse	113 (11)	65 (32)	48 (6)
SVR	892 (89)	140 (68)	752 (94)

SVR rates were calculated taking all patients with a documented 12-week FU after EoT into account. Values shown are counts and percentages, mean values ± standard deviation or median values with the corresponding IQR. Abbreviations: DAA, direct-acting antiviral; EoT, end of treatment; FU, follow-up; HCC, hepatocellular carcinoma; HCV, hepatitis C virus; HIV, human immunodeficiency virus; IFN, interferon; IQR, interquartile range; MELD, Model for End-Stage Liver Disease; SD, standard deviation; SVR, sustained virological response.

From October 2011 to March 2018, treatment numbers and patient characteristics changed due to the approval of new DAA regimens (S1 Table, treatment numbers by year). For example, while 71 HCV therapies were initiated in 2012, 300 therapies were initiated in 2015, and more patients were IFN-experienced in the earlier years (e.g. 55% in 2012 vs. 16% in 2017). Interestingly, the proportion of patients who were LTFU was higher over time (2/43, 5% in 2011 vs. 20/185, 11% in 2017).

With the introduction of each new generation of DAA regimens, SVR rates steadily improved from initially 60% and 67% with boceprevir and telaprevir therapy (S2 Table) [1], to SVR rates between 94% (sofosbuvir/ledipasvir) and 100% (glecaprevir/pibrentasvir) in later regimens (Table 2). The application of some of the earlier HCV DAA treatment regimens showed to be also very successful in specific cohorts (e.g. GT1b patients receiving elbasvir/grazoprevir: 18/18, SVR 100%; GT4 patients receiving ombitasvir/paritaprevir/ritonavir: 17/17, SVR 100%), and pangenotypic regimens proved to be highly successful across all GT. An overview of treatment numbers and SVR rates for each treatment regimen and GT are shown in S2 Table (IFN-based regimens) and Table 2 (IFN-free regimens).

**Table 2:** Baseline characteristics and treatment results in patients receiving an IFN-free DAA regimen.

IFN-free regimens	Complete cohort	GZR/EBR	GLE/PIB	OBV/PTV/r	OBV/PTV/r	SOF±RBV	SOF+DCV	SOF/LDV	SOF+SIM	SOF/VEL/	SOF/VEL
No of therapies	n = 878 (%)	n = 30 (%)	n = 47 (%)	n = 21 (%)	+DSV, n = 84 (%)	n = 49 (%)	n = 34 (%)	n = 449 (%)	n = 26 (%)	VOX, n = 14 (%)	n = 124 (%)
Age (y, mean ± SD)	53.1 ± 12.9	56.6 ± 14.4	50.0 ± 11.0	48.7 ± 13.7	59.2 ± 12.5	55.9 ± 11.2	55.3 ± 8.7	52.1 ± 13.2	57.1 ± 10.6	59.4 ± 11.3	50.1 ± 12.3
Sex (male/female)	486 (55) / 392 (45)	23 (77) / 7 (23)	26 (55) / 21 (45)	14 (66)/ 7 (33)	42 (50) / 42 (50)	28 (57) / 21 (43)	23 (68) / 11 (32)	237 (53) / 212 (47)	18 (69) / 8 (31)	11 (79) / 3 (21)	80 (65) / 44 (35)
Transplanted patients	54 (6)	1 (3)	0 (0)	0 (0)	0 (0)	14 (29)	5 (15)	22 (5)	9 (35)	1 (7)	2 (2)
People with HIV	38 (4)	1 (3)	0 (0)	0 (0)	1 (1)	4 (8)	5 (15)	15 (3)	0 (0)	0 (0)	12 (10)
Liver cirrhosis	254 (29)	12 (40)	1 (2)	2 (10)	23 (27)	13 (27)	22 (65)	117 (26)	18 (69)	8 (57)	38 (31)
Child-Pugh score A/B/C	180/71/3	9/3/0	1/0/0	2/0/0	21/2/0	8/5/0	12/9/1	87/29/1	9/9/0	3/5/0	28/9/1
IFN-experienced	267 (30)	1 (3)	5 (11)	4 (19)	21 (25)	28 (57)	18 (53)	147 (33)	14 (54)	6 (43)	23 (19)
HCV Genotype											
1 (a/b/c/unclassified)	616 [70] (270/327/3/16)	28 [93] (6/22/0/0)	25 [53] (16/8/0/1)	0 (0)	82 [98] (6/76/0/0)	7 [14] (2/5/0/0)	17 [50] (7/9/0/1)	414 [92] (209/190/2/13)	20 [77] (7/11/1/1)	9 [64] (6/3/0/0)	14 [11] (11/3/0/0)
2	44 (5)	0 (0)	6 (13)	0 (0)	0 (0)	25 (51)	0 (0)	0 (0)	0 (0)	2 (14)	11 (9)
3	148 (17)	0 (0)	10 (21)	0 (0)	0 (0)	12 (24)	15 (44)	14 (3)	2 (8)	2 (14)	93 (75)
4	58 (7)	2 (7)	4 (9)	21 (100)	1 (1)	3 (6)	2 (6)	17 (4)	4 (15)	1 (7)	3 (2)
5	2 (0)	0 (0)	1 (2)	0 (0)	0 (0)	1 (2)	0 (0)	0 (0)	0 (0)	0 (0)	0 (0)
6	3 (0)	0 (0)	0 (0)	0 (0)	0 (0)	0 (0)	0 (0)	3 (1)	0 (0)	0 (0)	0 (0)
1/3 coinfection	2 (0)	0 (0)	0 (0)	0 (0)	0 (0)	0 (0)	0 (0)	1 (0)	0 (0)	0 (0)	1 (1)
2k/1b	2 (0)	0 (0)	0 (0)	0 (0)	0 (0)	0 (0)	0 (0)	0 (0)	0 (0)	0 (0)	2 (2)
Unknown	3 (0)	0 (0)	1 (2)	0 (0)	1 (1)	1 (2)	0 (0)	0 (0)	0 (0)	0 (0)	0 (0)
**Outcome parameters**											
Lost to follow-up	78 (9)	6 (20)	1 (2)	4 (19)	5 (6)	2 (4)	1 (3)	48 (11)	1 (4)	0 (0)	10 (8)
Documented 12-wk FU after EoT	800 (91)	24 (80)	46 (98)	17 (81)	79 (94)	47 (96)	33 (97)	401 (89)	25 (96)	14 (100)	114 (92)
Virological relapse	48 (6)	1 (4)	0 (0)	0 (0)	2 (3)	8 (17)	6 (18)	23 (6)	5 (20)	1 (7)	2 (2)
SVR	752 (94)	23 (96)	46 (100)	17 (100)	77 (97)	39 (83)	27 (82)	378 (94)	20 (80)	13 (93)	112 (98)
GT 1a	212/231 (92)	3/4 (75)	16/16 (100)	n/a	4/5 (80)	2/2 (100)	3/6 (50)	168/178 (94)	4/6 (67)	5/6 (83)	7/8 (88)
GT 1b	295/306 (96)	18/18 (100)	8/8 (100)	n/a	71/72 (99)	3/5 (60)	9/9 (100)	171/177 (97)	9/11 (82)	3/3 (100)	3/3 (100)
GT 2	35/40 (88)	n/a	5/5 (100)	n/a	n/a	19/24 (79)	n/a	n/a	n/a	2/2 (100)	9/9 (100)
GT 3	136/142 (96)	n/a	10/10 (100)	n/a	n/a	10/11 (91)	12/15 (80)	12/14 (86)	2/2 (100)	2/2 (100)	88/89 (99)
GT 4	48/51 (94)	2/2 (100)	4/4 (100)	17/17 (100)	1/1 (100)	3/3 (100)	2/2 (100)	12/15 (80)	4/4 (100)	1/1 (100)	2/2 (100)
GT 5	2/2 (100)	n/a	1/1 (100)	n/a	n/a	1/1 (100)	n/a	n/a	n/a	n/a	n/a
GT 6	2/3 (67)	n/a	n/a	n/a	n/a	n/a	n/a	2/3 (67)	n/a	n/a	n/a

SVR rates were calculated taking all patients with a documented 12-week FU after EoT into account. Values shown are percentages and counts and mean values with the corresponding standard deviation.

Abbreviations: DAA, direct-acting antiviral; DCV, daclatasvir; DSV, dasabuvir; EBR, elbasvir; EoT, end of treatment; FU, follow-up; GLE, glecaprevir; GZR, grazoprevir; HCV, hepatitis C virus; HIV, human immunodeficiency virus; IFN, interferon; LDV, ledipasvir; n/a, not applicable; OBV, ombitasvir; PIB, pibrentasvir; PTV, paritaprevir; r, ritonavir; RBV, ribavirin; SD, standard deviation; SIM, simeprevir; SOF, sofosbuvir; SVR, sustained virological response; VEL, velpatasvir; VOX, voxilaprevir.

### Treatment results in patients with liver cirrhosis, HIV coinfection and liver transplanted patients

A relevant proportion of patients in our cohort were liver transplanted patients, patients with liver cirrhosis or people with HIV. Baseline characteristics of these special cohorts were generally comparable to the overall cohort (S3 Table). The relative LTFU rate was especially low (3%) in liver transplant recipients, but similar in comparison to the overall cohort in patients with liver cirrhosis and people with HIV (8% each, respectively). SVR rates were also comparable in these special cohorts, especially for the IFN-free DAA regimens reaching up to 100% for various combination therapies. In line with that, a previous liver transplantation, liver cirrhosis or an HIV coinfection were not associated with treatment failure on multivariable analysis (Table 3). However, the treatment response was lower in patients with liver cirrhosis and HCC, with an SVR rate of 65% (S3 Table). Accordingly, the presence of an HCC was also associated with treatment failure on multivariable analysis (Table 3).

**Table 3:** Uni- and multivariable logistic regression to identify factors associated with a virological relapse.

			95%-confidence interval for odds ratio	
Parameter	p-value	Odds ratio	Lower	Upper
**Univariable logistic regression**				
Female sex	0.003	1.915	1.265	2.956
Age	0.743	0.999	0.995	1.008
HIV coinfection	0.607	1.316	0.519	4.442
Prior liver transplantation	0.640	0.832	0.406	1.936
Presence of HCC	0.001	0.226	0.096	0.572
IFN treatment experience	<0.001	0.242	0.158	0.366
DAA treatment experience	<0.001	0.330	0.206	0.540
Liver cirrhosis at baseline	<0.001	0.257	0.171	0.383
Genotype 1a	0.001	0.506	0.340	0.757
Genotype 1b	0.392	1.195	0.799	1.811
Genotype 2	0.798	0.882	0.369	2.612
Genotype 3	0.006	2.847	1.445	6.461
Genotype 4	0.212	1.930	0.775	6.452
Other genotype	0.320	0.665	0.302	1.638
IFN-based DAA therapy	<0.001	0.136	0.090	0.208
Liver stiffness at baseline	<0.001	0.949	0.934	0.963
**Multivariable logistic regression**				
Female sex	0.916	0.964	0.490	1.918
Presence of HCC	0.032	0.077	0.007	0.904
IFN treatment experience	0.010	0.396	0.194	0.798
DAA treatment experience	0.159	0.541	0.234	1.303
Liver cirrhosis at baseline	0.854	1.088	0.454	2.739
Genotype 1a	0.412	0.752	0.382	1.499
Genotype 3	0.150	2.311	0.801	8.128
IFN-based DAA therapy	<0.001	0.111	0.054	0.218
Liver stiffness at baseline	<0.001	0.940	0.915	0.964

All parameters with a p-value <0.1 in the univariable logistic regression were included in the multivariable logistic regression. Abbreviations: DAA, direct-acting antiviral; HCC, hepatocellular carcinoma; HIV, human immunodeficiency virus; IFN, interferon; SVR, sustained virological response.

### Virological relapse and re-treatment regimens

Patients experiencing a virological relapse were predominantly male (70/117, 60%) and more likely to have a liver cirrhosis at baseline compared to the complete cohort (51/117, 44% vs. 29%). Most patients experienced a single relapse (97/117, 83%), but relapses after two (18/117, 15%) or even three (2/117, 2%) treatment courses were also observed, resulting in a total of 139 individual therapies. In univariable analysis, female sex (odds ratio [OR] 1.915, 95%-confidence interval [CI] 1.265–2.956), presence of an HCC (OR 0.226, 95%-CI 0.096–0.572), IFN treatment experience (OR 0.242, 95%-CI 0.158–0.366), DAA treatment experience (OR 0.330, 95%-CI 0.206–0.540), liver cirrhosis at baseline (OR 0.257, 95%-CI 0.171–0.383), GT1a (OR 0.506, 95%-CI 0.340–0.757), GT3 (OR 2.847, 95%-CI 1.445–6.461), IFN-based DAA therapy (OR 0.136, 95%-CI 0.090–0.208) and LS at baseline (OR 0.949, 95%-CI 0.934–0.963) were associated with treatment failure. In multivariable analysis, presence of an HCC (OR 0.077, 95%-CI 0.007–0.904), IFN treatment experience (OR 0.396, 95%-CI 0.194–0.798), an IFN-based therapy (OR 0.111; 95%-CI 0.054–0.218) and LS at baseline (OR 0.940, 95%-CI 0.915–0.964) were associated with treatment failure, whereas DAA treatment experience (OR 0.541; 95%-CI 0.234–1.303) or a specific GT were not risk factors in this cohort (Table 3).

A relapse was predominantly observed after treatment with an IFN-based protease inhibitor-containing regimen (boceprevir: 22/139, 16%; telaprevir: 27/139, 19%; clinical trial regimen: 19/139, 14%). In these patients, different re-treatment strategies were used, but in general, a switch of drug class and therefore re-treatment with a NS5A or NS5B inhibitor–or the combination of both–achieved high SVR rates (e.g. sofosbuvir/ledipasvir after boceprevir: 9/10, 90% SVR; after telaprevir: 12/13, 92% SVR; after clinical trial protease inhibitor-containing regimen: 11/12, 91% SVR). Even though less frequently used, re-treatment with other IFN-free combination regimens also yielded excellent results (Table 4).

**Table 4:** Baseline characteristics and re-treatment regimens in patients with a previous failure to an IFN-based DAA regimen.

Failure of	BOC n = 22 (%)	TVR n = 27 (%)	DCV n = 3 (%)	SOF+IFN n = 16 (%)	SIM n = 1 (%)	PI clinical trial n = 19 (%)
Age (y, mean ± SD)	54.0 ± 12.1	49.6 ± 12.8	52.9 ± 14.5	56.7 ± 8.0	65	52.6 ± 13.6
Sex (male/female)	11 (50) / 11 (50)	16 (59) / 11 (41)	2 (67) / 1 (33)	11 (69) / 5 (31)	1 (100) / 0 (0)	8 (42) / 11 (58)
Liver transplanted patients	1 (5)	3 (11)	0 (0)	2 (13)	0 (0)	0 (0)
People with HIV	0 (0)	2 (7)	0 (0)	1 (6)	0 (0)	0 (0)
Liver cirrhosis	7 (32)	12 (44)	1 (33)	9 (56)	1 (100)	4 (21)
Child-Pugh score A/B/C	7/0/0	9/3/0	1/0/0	7/2/0	1/0/0	3/1/0
IFN-experienced	16 (73)	22 (81)	1 (33)	15 (94)	1 (100)	12 (63)
HCV Genotype						
1 (a/b/c/unclassified)	22 [100] (15/6/0/1)	27 [100] (13/12/1/1)	3 [100] (1/2/0/0)	13 [81] (3/10/0/0)	1 [100] (1/0/0/0)	15 [79](5/9/0/1)
2	0 (0)	0 (0)	0 (0)	0 (0)	0 (0)	0 (0)
3	0 (0)	0 (0)	0 (0)	1 (6)	0 (0)	3 (16)
4	0 (0)	0 (0)	0 (0)	1 (6)	0 (0)	1 (5)
5	0 (0)	0 (0)	0 (0)	0 (0)	0 (0)	0 (0)
6	0 (0)	0 (0)	0 (0)	1 (6)	0 (0)	0 (0)
**Re-treatment with**						
No re-treatment	5 (23)	3 (11)	0 (0)	2 (13)	0 (0)	0 (0)
BOC [SVR%]	n/a	2 (7) / 50%	n/a	n/a	n/a	n/a
TVR [SVR%]	1 (5) / 100%	n/a	2 (67) / 50%	n/a	n/a	2 (11) / 50%
SOF+IFN [SVR%]	2 (9) / 100%	5 (19) / 0%	n/a	n/a	n/a	1 (5) / 100%
SOF+RBV [SVR%]	1 (5) / 100%	1 (4) / 0%	n/a	n/a	n/a	n/a
SOF+SIM [SVR%]	n/a	2 (7) / 50%	n/a	n/a	n/a	n/a
SOF+DCV [SVR%]	1 (5) / 100%	1 (4) / 0%	n/a	n/a	1 (100) / 0%	n/a
OBV/PTV/r+DSV [SVR%]	1 (5) / 100%	n/a	1 (33) / 100%	4 (25) / 100%	n/a	n/a
GLE/PIB [SVR%]	1 (5) / 100%	n/a	n/a	n/a	n/a	n/a
SOF/LDV [SVR%]	10 (45) / 90%	13 (48) / 92%	n/a	10 (63) / 70%	n/a	12 (63) / 91%
SOF/VEL [SVR%]	n/a	n/a	n/a	n/a	n/a	3 (16) / 100%
SOF/VEL/VOX [SVR%]	n/a	n/a	n/a	n/a	n/a	1 (5) / 100%

Baseline characteristics of patients with treatment failure to a specific IFN-based DAA regimen are depicted in the upper part of the table, the lower part of the table shows the respective re-treatment regimens. Next to each re-treatment regimen, the absolute and relative number of patients treated with each regimen is shown, followed by the respective SVR rate (read: n (%) / SVR%). Values shown are counts and percentages or mean values ± standard deviation.

Abbreviations: BOC, boceprevir; DAA, direct-acting antiviral; DCV, daclatasvir; DSV, dasabuvir; GLE, glecaprevir; HCV, hepatitis C virus; HIV, human immunodeficiency virus; IFN, interferon; LDV, ledipasvir; n/a, not applicable; OBV, ombitasvir; PI, protease inhibitor; PIB, pibrentasvir; PTV, paritaprevir; r, ritonavir; RBV, ribavirin; SD, standard deviation; SIM, simeprevir; SOF, sofosbuvir; SVR, sustained virological response; TVR, telaprevir; VEL, velpatasvir; VOX, voxilaprevir.

Treatment failure after an IFN-free DAA regimen occurred in 51/139 patients (37%) and mainly after treatment with sofosbuvir+ribavirin (10/139, 7%) and the most frequently used combination regimen sofosbuvir/ledipasvir (23/139, 17%). Of note, the proportion of patients that had not received re-treatment was higher in patients initially receiving an IFN-free DAA regimen compared to patients that had initially received an IFN-based DAA therapy (17/51, 33% vs. 10/88, 11%).

**Fig 1:**
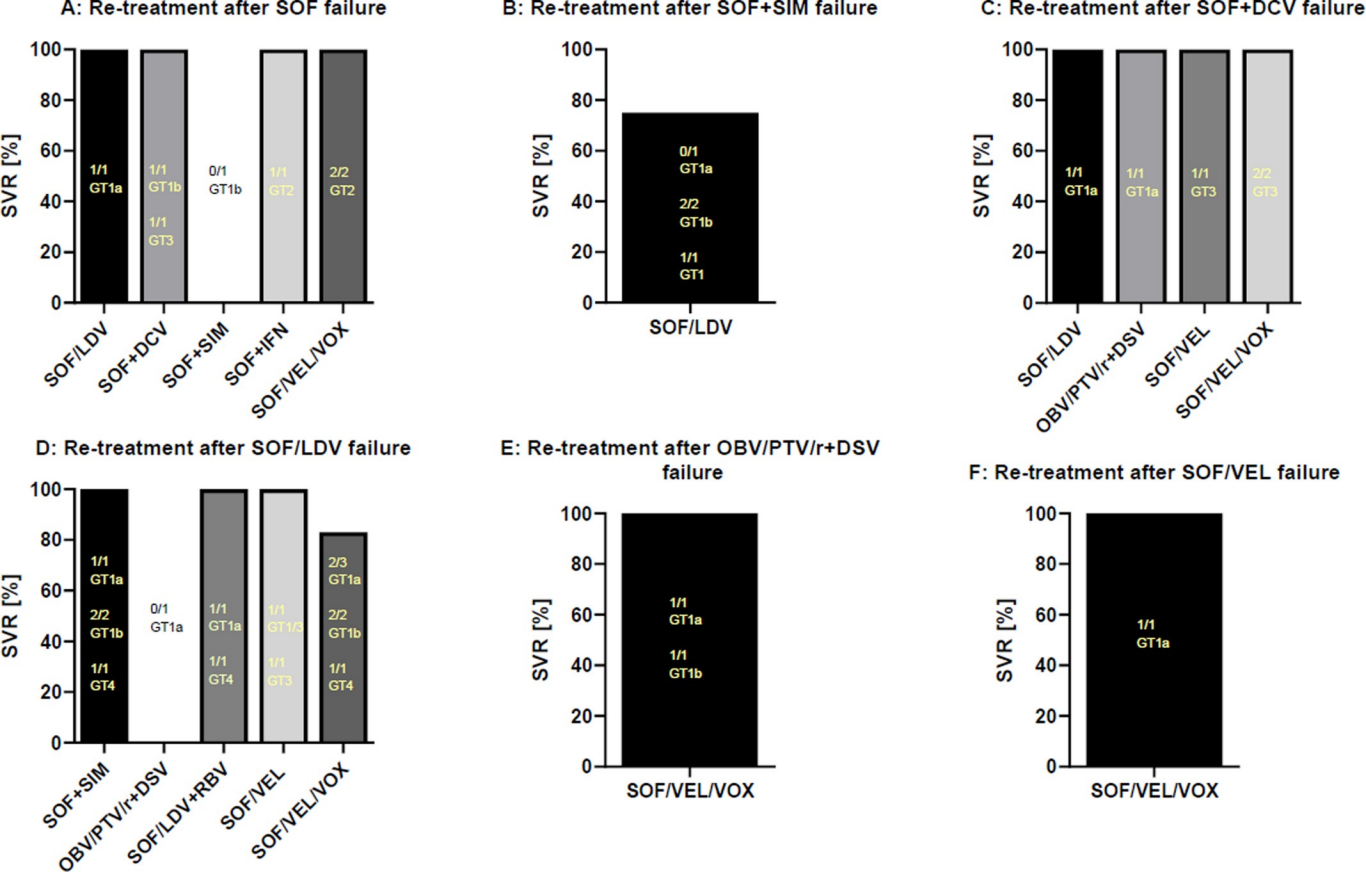
Re-treatment regimens after failure of an IFN-free DAA therapy. Re-treatment regimens used after failure of a first IFN-free DAA therapy with SOF (A), SOF+SIM (B) SOF+DCV (C), SOF/LDV (D), OBV/PTV/r+DSV (E) and SOF/VEL (F) are shown. Abbreviations: DAA, direct-acting antiviral; DCV, daclatasvir; DSV, dasabuvir; GT, genotype; IFN, interferon; LDV, ledipasvir; OBV, ombitasvir; PTV, paritaprevir; r, ritonavir; RBV, ribavirin; SIM, simeprevir; SOF, sofosbuvir; SVR, sustained virological response; VEL, velpatasvir; VOX, voxilaprevir.

After an unsuccessful treatment course with sofosbuvir/ledipasvir, re-treatment with sofosbuvir/velpatasvir/voxilaprevir (5/6, SVR 83%), sofosbuvir/velpatasvir (2/2, SVR 100%) and sofosbuvir/simeprevir in GT1 and GT4 patients (4/4, SVR 100%) achieved excellent results in this small patient sample. Additionally, sofosbuvir/velpatasvir/voxilaprevir was the most commonly used regimen after failure of an DAA-based treatment regimen with an overall high SVR rate of 93% (13/14, see Table 2). In total, 82 of 96 (85%) re-treatment attempts with all-oral DAA regimens were successful after an initial treatment failure. Fig 1 and Table 5 give a detailed overview of re-treatment regimens after previous failure of a first IFN-free treatment course.

**Table 5:** Baseline characteristics and re-treatment regimens in patients with a previous failure to an IFN-free DAA regimen.

Failure of	SOF n = 10 (%)	SOF+SIM n = 5 (%)	SOF+DCV n = 7 (%)	SOF/LDV n = 23 (%)	OBV/PTV/r+ DSV, n = 2 (%)	SOF/VEL n = 2 (%)	GZR/EBR n = 1 (%)	SOF/VEL/VOX n = 1 (%)
Age (y, mean ± SD)	54.4 ± 12.9	59.4 ± 4.6	54.6 ± 11.4	57.5 ± 8.8	66.5 ± 12.7	43.9 ± 10.1	34	63
Sex (male/female)	6 (60) / 4 (40)	4 (80) / 1 (20)	7 (100) / 0 (0)	19 (83) / 4 (17)	1 (50) / 1 (50)	1 (50) / 1 (50)	0 (0) / 1 (100)	1 (100) / 0 (0)
Liver transplanted patients	2 (20)	0 (0)	0 (0)	0 (0)	0 (0)	0 (0)	0 (0)	0 (0)
People with HIV	0 (0)	0 (0)	0 (0)	1 (4)	0 (0)	0 (0)	0 (0)	0 (0)
Liver cirrhosis	4 (40)	5 (100)	4 (57)	19 (83)	2 (100)	2 (100)	0 (0)	1 (100)
Child-Pugh score A/B/C	2/2/0	1/4/0	4/0/0	8/11/0	2/0/0	1/1/0	n/a	0/1/0
IFN-experienced	7 (70)	3 (60)	4 (57)	9 (39)	1 (50)	0 (0)	0 (0)	0 (0)
HCV Genotype								
1 (a/b/unclassified)	4 [40] (1/3/0)	5 [100] (2/2/1)	3 [43] (3/0/0)	16 [70] (10/6/0)	2 [100] (1/1/0)	1 [50] (1/0/0)	1 [100] (0/1/0)	1 [100] (1/0/0)
2	5 (50)	0 (0)	0 (0)	0 (0)	0 (0)	0 (0)	0 (0)	0 (0)
3	1 (10)	0 (0)	4 (57)	2 (9)	0 (0)	1 (50)	0 (0)	0 (0)
4	0 (0)	0 (0)	0 (0)	3 (13)	0 (0)	0 (0)	0 (0)	0 (0)
5	0 (0)	0 (0)	0 (0)	0 (0)	0 (0)	0 (0)	0 (0)	0 (0)
6	0 (0)	0 (0)	0 (0)	1 (4)	0 (0)	0 (0)	0 (0)	0 (0)
1/3 coinfection	0 (0)	0 (0)	0 (0)	1 (4)	0 (0)	0 (0)	0 (0)	0 (0)
**Re-treatment with**								
No re-treatment	3 (30)	1 (20)	2 (29)	8 (35)	0 (0)	1 (50)	1 (100)	1 (100)
SOF+IFN [SVR%]	1 (10) / 100%	n/a	n/a	n/a	n/a	n/a	n/a	n/a
SOF+SIM [SVR%]	1 (10) / 0%	n/a	n/a	4 (17) / 100%	n/a	n/a	n/a	n/a
SOF+DCV [SVR%]	2 (20) / 100%	n/a	n/a	n/a	n/a	n/a	n/a	n/a
OBV/PTV/r+DSV [SVR%]	n/a	n/a	1 (14) / 100%	1 (4) / 0%	n/a	n/a	n/a	n/a
SOF/LDV [SVR%]	1 (10) / 100%	4 (80) / 75%	1 (14) / 100%	n/a	n/a	n/a	n/a	n/a
SOF/LDV+RBV [SVR%]	n/a	n/a	n/a	2 (9) / 100%	n/a	n/a	n/a	n/a
SOF/VEL [SVR%]	n/a	n/a	1 (14) / 100%	2 (9) / 100%	n/a	n/a	n/a	n/a
SOF/VEL/VOX [SVR%]	2 (20) / 100%	n/a	2 (28) / 100%	6 (26) / 83%	2 (100) / 100%	1 (50) / 100%	n/a	n/a

Baseline characteristics of patients with treatment failure to a specific IFN-free DAA regimen are depicted in the upper part of the table, the lower part of the table shows the respective re-treatment regimens. Next to each re-treatment regimen, the absolute and relative number of patients treated with each regimen is shown, followed by the respective SVR rate (read: n (%) / SVR%). Values shown are counts and percentages or mean values ± standard deviation.

Abbreviations: DAA, direct-acting antiviral; DCV, daclatasvir; DSV, dasabuvir; EBR, elbasvir; GLE, glecaprevir; GZR, grazoprevir; HCV, hepatitis C virus; HIV, human immunodeficiency virus; IFN, interferon; LDV, ledipasvir; n/a, not applicable; OBV, ombitasvir; PIB, pibrentasvir; PTV, paritaprevir; r, ritonavir; RBV, ribavirin; SD, standard deviation; SIM, simeprevir; SOF, sofosbuvir; SVR, sustained virological response; VEL, velpatasvir; VOX, voxilaprevir.

### RAS testing

Baseline RAS testing was only available in a subgroup of 67 DAA treatment-naïve patients prior to DAA therapy and in a subgroup of 36 patients after virological relapse and prior to re-treatment. While RAS were only detected in 11/67 (11%) of treatment-naïve patients, numbers were much higher in treatment-experienced patients (29/36, 81%). Most RAS were observed in the NS5A-gene, including the high-level resistances Y93H and L31M, but did not seem to affect re-treatment outcome since SVR rates were equally high in both treatment-naïve and treatment-experienced patients (S4 Table).

## Discussion

In this study, we present an overview of almost 1100 DAA-based therapies in different patient cohorts, including the sequential treatment and re-treatment results in a real-world setting. Thus, our study summarizes the real-world use and success rate of each DAA regimen that has been introduced for HCV treatment since 2011.

Treatment numbers increased at our center after the introduction of sofosbuvir and sofosbuvir/ledipasvir in 2014, also improving treatment results with yearly SVR rates of 98% in 2016 and 2017. Furthermore, treatment-naïve patients predominated our cohort in the later years, and the rate of LTFU patients also increased, which might be explained with lower treatment barriers and less stringent monitoring requirements due to improvements in the security and side effect profiles of each new generation of DAA regimens. In addition, our data also confirm that especially IFN-free DAA treatment regimens are just as effective in formerly difficult to treat patient cohorts like liver transplant recipients and people with HIV in a real-world setting [4]. On the other hand, as reported before [17], treatment success was lower in patients with liver cirrhosis and HCC in our cohort, so that the presence of an HCC was also a risk factor for treatment failure in the multivariable analysis.

Importantly, we also assessed the success of re-treatment regimens, as data on optimized DAA re-treatment strategies after virological failure of other DAA-containing therapies remain scarce. Thus, data from the current and other real-world studies add to a growing body of evidence to formulate optimized and evidence-based re-treatment recommendations [9]. Especially after unsuccessful treatment with an IFN-based protease inhibitor regimen, our data indicate that a switch to–or addition of–a new drug class and thus re-treatment with any fixed-dose IFN-free treatment regimen is a feasible and successful strategy, an observation that has also been reported by other studies [10, 18, 19] and is supported by current guidelines [9]. Thus, in these patients, the usage of sofosbuvir/velpatasvir/voxilaprevir, the only drug explicitly approved for re-treatment after DAA failure, may not be always necessary, as its availability and remaining high cost could limit providers from its broader application.

With regard to the re-treatment of patients with a previous failure to an IFN-free treatment regimen, our experience also support that a change in drug class–e.g. re-treatment with sofosbuvir/simeprevir after failure of sofosbuvir/ledipasvir–seems to be an alternative, which has also been reported in different other cohorts [5, 20, 21]. In this context, the use of sofosbuvir/velpatasvir merits further discussion, since other reports have shown excellent treatment results [2, 22] including an SVR rate of 90% in the POLARIS-4 trial [10]. In line with this, the three patients in our cohort who received this regimen for re-treatment also achieved an SVR–even though our patient numbers alone are too small to draw a general conclusion. Still, the most frequently used DAA re-treatment option at our center was sofosbuvir/velpatasvir/voxilaprevir, which yielded excellent results despite the difficult to treat population, including the presence of baseline RAS. In this context, larger studies need to clarify the role of RAS testing before the initiation of DAA re-treatment [23, 24], as RAS did not seem to affect re-treatment results in our small sample. This is especially interesting as RAS testing was mostly carried out in patients in whom RAS were *a priori* hypothesized to be relevant. Therefore, even though helpful for certain case constellations, the HCV re-treatment concept of switching or adding a different drug class independent of RAS testing (e.g. in centers with limited access to RAS testing) seems to be a valid concept.

Furthermore, only two patients failed more than two DAA treatment courses, of which one patient received treatment after placement of a transjugular intrahepatic portosystemic shunt (TIPS). Therefore, we hypothesize that failing multiple treatments might be limited to a certain subgroup of patients with a combination of unfavorable baseline characteristics like decompensated liver cirrhosis, extensive RAS or previous TIPS placement [25]. Therefore, an individualized treatment concept that takes all individual risk factors into account is warranted in these patients. In addition, 33% of patients with a previous failure to an IFN-free DAA regimen had no documented re-treatment in our cohort, suggesting that these patients should be identified and informed about the now available re-treatment options.

Despite describing treatment outcomes in a relatively large cohort, this study has several limitations due to its retrospective nature. For one, treatment duration and additional ribavirin usage were at the discretion of each individual provider. Furthermore, even though the choice of re-treatment regimen was mostly made after the consultation of an internal hepatitis board, a broad variety of different regimens has been used, especially after failure of an IFN-free DAA regimen. As a consequence, the numbers for each re-treatment regimen presented in this manuscript after previous DAA treatment failure are too small to draw conclusions. Due to the small numbers, we were also unable to provide more information on re-treatment results in patients who had previously failed treatment attempts with two IFN-free DAA regimens. As this is an issue that has not been properly assessed in the literature, larger studies are needed in this regard.

This current analysis illustrates nicely the evolution of HCV treatment at a German center from 2011 until now, starting with the introduction of the first-generation protease inhibitors. At all times, treatment decisions were guided by availability, guidelines and cost-effectiveness–therefore, this retrospective study also illustrates how certain weaknesses of earlier regimens influenced later treatment decisions. Even though a little academic, musing on how many patients would have profited more from a “wait and see” strategy (i.e. to withhold treatment until the introduction of the best fitting DAA regimen) rather than being treated with a regimen at hand at that specific timepoint is an interesting mind game. Therefore, it will be interesting to compare our results–that certainly only reflect the local situation–with treatment and re-treatment results in regions of the world that have no or little access to RAS testing or DAA rescue regimens [26, 27].

In conclusion, this descriptive analysis confirms that DAA treatment is highly effective and that a virological relapse only occurs in very few patients with modern and highly potent regimens. Treatment failure to two all-oral DAA treatment regimens are also rarely observed, and our data suggest that a switch to–or addition of–a new drug class are good re-treatment options.

## Supporting information

S1 Table Treatment numbers and SVR rates for each year since the introduction of DAA-based HCV therapies. SVR rates were calculated taking all patients with a documented 12-week FU after EoT into account. Values shown are percentages and counts and mean or median values with the corresponding standard deviation or IQR. Abbreviations: DAA, direct acting antiviral; EoT, end of treatment; FU, follow-up; HCV; hepatitis C virus; HIV, human immunodeficiency virus; IFN, interferon; IQR, interquartile range; MELD, Model for End-Stage Liver Disease; SD, standard deviation; SVR, sustained virological response.
(DOCX)

S2 Table Baseline characteristics and treatment results in patients receiving an IFN-based DAA regimen. SVR rates were calculated taking all patients with a documented 12-week FU after EoT into account. Values shown are percentages and counts and mean values with the corresponding standard deviation. Abbreviations: EoT, end of treatment; FU, follow-up; DAA, direct acting antiviral; GT, genotype; HCV, hepatitis C virus; HIV, human immunodeficiency virus; IFN, interferon; SD, standard deviation.
(DOCX)

S3 Table Baseline characteristics and treatment results in special cohorts. Baseline characteristics of each special cohort are depicted in the upper part of the table, the lower part of the table shows the outcome of treatment regimens. Next to each treatment regimen in the lower part of the table, the absolute and relative number of patients treated with each regimen is shown, followed by the respective SVR rate (read: n (%) / SVR%). SVR rates were calculated only in patients with a documented 12-week FU after EoT. Values shown are percentages and counts and mean or median values with the corresponding standard deviation or IQR. Abbreviations: BOC, boceprevir; DAA, direct-acting antiviral; DCV, daclatasvir; DSV, dasabuvir; EBR, elbasvir; EoT, end of treatment; FU, follow-up; GLE, glecaprevir; GZR, grazoprevir; HCV, hepatitis C virus; HIV, human immunodeficiency virus; IFN, interferon; IQR, interquartile range; LDV, ledipasvir; MELD, Model for End-Stage Liver Disease; n/a, not applicable; OBV, ombitasvir; PIB, pibrentasvir; PTV, paritaprevir; r, ritonavir; RBV, ribavirin; SD, standard deviation; SIM, simeprevir; SOF, sofosbuvir; SVR, sustained virological response; TVR, telaprevir; VEL, velpatasvir; VOX, voxilaprevir.
(DOCX)

S4 Table RAS and SVR rates of first line or re-treatment regimens. Abbreviations: DAA, direct-acting antiviral; DCV, daclatasvir; DSV, dasabuvir; EBR, elbasvir; GLE, glecaprevir; GT, genotype; GZR, grazoprevir; RAS, resistance-associated substitution; LDV, ledipasvir; n/a, not applicable; OBV, ombitasvir; PIB, pibrentasvir; PTV, paritaprevir; r, ritonavir; RBV, ribavirin; SIM, simeprevir; SOF, sofosbuvir; SVR, sustained virological response; VEL, velpatasvir; VOX, voxilaprevir.
(DOCX)

## Abbreviations

CI: confidence interval
DAA: direct-acting antiviral
EoT: end of treatment
FU: follow-up
GT: genotype
HCC: hepatocellular carcinoma
HCV: hepatitis C virus
HIV: human immunodeficiency virus
IFN: interferon
IQR: interquartile range
LS: liver stiffness
LTFU: lost to follow-up
OR: odds ratio
RAS: resistance-associated substitution
SD: standard deviation
SVR: sustained virological response
TE: transient elastography
TIPS: transjugular intrahepatic portosystemic shunt
