# Supplementary material for: Treatment and re-treatment results of HCV patients in the DAA era
Source: PLoS One. 2020 May 5;15(5):e0232773. doi: 10.1371/journal.pone.0232773 (PMC7200014; doi:10.1371/journal.pone.0232773)
Supplement: S1 Table — SVR rates were calculated taking all patients with a documented 12-week FU after EoT into account. Values shown are percentages and counts and mean or median values with the corresponding standard deviation or IQR. Abbreviations: DAA, direct acting antiviral; EoT, end of treatment; FU, follow-up; HCV; hepatitis C virus; HIV, human immunodeficiency virus; IFN, interferon; IQR, interquartile range; MELD, Model for End-Stage Liver Disease; SD, standard deviation; SVR, sustained virological response. (DOCX) [file pone.0232773.s001.docx]

| **No of therapies** | **Complete cohort**  **n=1096 (%)** | **2011**  **n=43 (%)** | **2012**  **n=71 (%)** | **2013**  **n=26 (%)** | **2014**  **n=271 (%)** | **2015**  **n=300 (%)** | **2016**  **n=179 (%)** | **2017**  **n=185 (%)** | **Until 03/2018**  **n=21 (%)** |
| --- | --- | --- | --- | --- | --- | --- | --- | --- | --- |
| Age (y, mean ± SD) | 52.3 ± 12.7 | 47.3 ± 13.4 | 49.1 ± 10.8 | 51.5 ± 12.4 | 53.8 ± 11.4 | 52.8 ± 13.2 | 53.3 ± 13.3 | 51.3 ± 13.6 | 51.0 ± 11.4 |
| Sex (male/female) | 628 (57) / 468 (43) | 24 (56) / 19 (44) | 45 (63) / 26 (37) | 14 (54) / 12 (46) | 167 (62) / 104 (38) | 166 (55) / 134 (45) | 98 (57) / 81 (45) | 98 (53) / 87 (47) | 16 (76) / 5 (24) |
| Liver transplanted patients | 63 (6) | 0 (0) | 3 (4) | 5 (19) | 35 (13) | 11 (4) | 5 (3) | 4 (2) | 0 (0) |
| People with HIV | 49 (4) | 2 (5) | 3 (4) | 2 (8) | 10 (4) | 15 (5) | 7 (4) | 9 (5) | 1 (5) |
| Liver cirrhosis  Child-Pugh score A/B/C  Median MELD (IQR) | 320 (29)  238/79/3  8.0 (7.0, 10.5) | 14 (33)  13/1/0  8.0 (6.5, 9.0) | 20 (28)  17/3/0  7.0 (7.0, 8.5) | 6 (23)  5/1/0  5.0 (5.0, 7.0) | 106 (39)  70/36/0  6.0 (5.0, 7.0) | 81 (27)  66/14/1  8.0 (7.0, 10.0) | 35 (20)  28/6/1  5.0 (5.0, 6.0) | 53 (29)  37/15/1  6.0 (5.0, 7.0) | 5 (24)  2/3/0  7.0 (5.0, 7.0) |
| IFN-experienced | 384 (35) | 21 (49) | 39 (55) | 20 (77) | 131 (50) | 36 (12) | 41 (23) | 29 (16) | 1 (5) |
| HCV Genotype  1 (a/b/c/unclassified)  2  3  4  5  6  1/3 coinfection  2k/1b  Unknown | 791 [72] (342/418/5/26)  44 (4)  173 (16)  71 (6)  3 (0)  6 (1)  2 (0)  2 (0)  4 (0) | 43 [100] (15/23/0/5)  0 (0)  0 (0)  0 (0)  0 (0)  0 (0)  0 (0)  0 (0)  0 (0) | 70 [99] (28/39/0/3)  0 (0)  0 (0)  0 (0)  0 (0)  0 (0)  0 (0)  0 (0)  1 (1) | 26 [100] (13/11/2/0)  0 (0)  0 (0)  0 (0)  0 (0)  0 (0)  0 (0)  0 (0)  0 (0) | 187 [69] (82/95/2/8)  15 (6)  39 (14)  25 (9)  1 (0)  3 (1)  0 (0)  0 (0)  1 (0) | 244 [81] (96/141/1/6)  7 (2)  26 (9)  19 (7)  1 (0)  2 (1)  1 (0)  0 (0)  0 (0) | 100 [56] (40/58/0/2)  13 (7)  51 (28)  14 (8)  0 (0)  0 (0)  0 (0)  0 (0)  1 (1) | 111 [60] (61/49/0/1)  6 (4)  51 (28)  12 (6)  1 (1)  1 (1)  1 (1)  1 (1)  1 (1) | 10 [48] (8/2/0/0)  3 (14)  6 (29)  1 (5)  0 (0)  0 (0)  0 (0)  1 (5)  0 (0) |
| **Outcome parameters** |  |  |  |  |  |  |  |  |  |
| Lost to follow-up | 91 (8) | 2 (5) | 4 (6) | 2 (8) | 18 (7) | 26 (9) | 19 (11) | 20 (11) | 0 (0) |
| Documented 12-wk FU after EoT | 1005 (92) | 41 (95) | 67 (94) | 24 (92) | 253 (93) | 274 (91) | 160 (89) | 165 (89) | 21 (100) |
| Virological relapse | 113 (11) | 11 (27) | 22 (33) | 14 (58) | 42 (17) | 16 (6) | 4 (3) | 4 (2) | 0 (0) |
| SVR | 892 (89) | 30 (73) | 45 (67) | 10 (42) | 211 (83) | 258 (94) | 156 (98) | 161 (98) | 21 (100) |

**S1 Table. Treatment numbers and SVR rates for each year since the introduction of DAA-based HCV therapies**

Table legend: SVR rates were calculated taking all patients with a documented 12-week FU after EoT into account. Values shown are percentages and counts and mean or median values with the corresponding standard deviation or IQR.

Abbreviations: DAA, direct acting antiviral; EoT, end of treatment; FU, follow-up; HCV; hepatitis C virus; HIV, human immunodeficiency virus; IFN, interferon; IQR, interquartile range; MELD, Model for End-Stage Liver Disease; SD, standard deviation; SVR, sustained virological response
