# Supplementary material for: Treatment and re-treatment results of HCV patients in the DAA era
Source: PLoS One. 2020 May 5;15(5):e0232773. doi: 10.1371/journal.pone.0232773 (PMC7200014; doi:10.1371/journal.pone.0232773)
Supplement: S2 Table — SVR rates were calculated taking all patients with a documented 12-week FU after EoT into account. Values shown are percentages and counts and mean values with the corresponding standard deviation. Abbreviations: EoT, end of treatment; FU, follow-up; DAA, direct acting antiviral; GT, genotype; HCV, hepatitis C virus; HIV, human immunodeficiency virus; IFN, interferon; SD, standard deviation. (DOCX) [file pone.0232773.s002.docx]

**S2 Table.** **Baseline characteristics and treatment results in patients receiving an IFN-based DAA regimen**

| **IFN-based regimens**  **No of therapies** | **Complete cohort**  **n=218 (%)** | **Boceprevir**  **n=50 (%)** | **Telaprevir**  **n=86 (%)** | **Daclatasvir**  **n=4 (%)** | **Sofosbuvir**  **n=77 (%)** | **Simeprevir**  **n=1 (%)** |
| --- | --- | --- | --- | --- | --- | --- |
| Age (y, mean ± SD) | 49.3 ± 11.8 | 50.7 ± 12.0 | 48.3 ± 11.8 | 42.0 ± 14.6 | 49.7 ± 11.4 | 65 |
| Sex (male/female) | 142 (65) / 76 (35) | 29 (58) / 21 (42) | 53 (62) / 33 (38) | 3 (75) / 1 (25) | 56 (73) / 21 (27) | 1 (100) / 0 (0) |
| Liver transplanted patients | 9 (4) | 2 (4) | 5 (6) | 0 (0) | 2 (3) | 0 (0) |
| People with HIV | 11 (5) | 2 (4) | 5 (6) | 0 (0) | 4 (5) | 0 (0) |
| Liver cirrhosis  Child-Pugh score A/B/C | 66 (30)  58/8/0 | 9 (18)  9/0/0 | 30 (35)  25/5/0 | 1 (25)  1/0/0 | 25 (32)  22/3/0 | 1 (100)  1/0/0 |
| IFN-experienced | 117 (54) | 25 (50) | 53 (62) | 1 (25) | 37 (48) | 1 (100) |
| HCV Genotype  1 (a/b/c/unclassified)  2  3  4  5  6  1/3 coinfection  2k/1b  Unknown | 175 [80] (73/91/2/9)  0 (0)  25 (11)  13 (6)  1 (0)  3 (1)  0 (0)  0 (0)  1 (0) | 50 [100] (24/23/0/3)  0 (0)  0 (0)  0 (0)  0 (0)  0 (0)  0 (0)  0 (0)  0 (0) | 85 [99] (31/47/1/5)  0 (0)  0 (0)  0 (0)  0 (0)  0 (0)  0 (0)  0 (0)  1 (1) | 4 [100] (1/3/0/0)  0 (0)  0 (0)  0 (0)  0 (0)  0 (0)  0 (0)  0 (0)  0 (0) | 35 [45] (16/18/1/0)  0 (0)  25 (32)  13 (17)  1 (1)  3 (4)  0 (0)  0 (0)  0 (0) | 1 [100] (1/0/0/0)  0 (0)  0 (0)  0 (0)  0 (0)  0 (0)  0 (0)  0 (0)  0 (0) |
| **Outcome parameters** |  |  |  |  |  |  |
| Lost to follow-up | 13 (6) | 0 (0) | 8 (9) | 0 (0) | 5 (6) | 0 (0) |
| Documented 12-wk FU after EoT | 205 (94) | 50 (100) | 78 (91) | 4 (100) | 72 (94) | 1 (100) |
| Virological relapse | 65 (32) | 20 (40) | 26 (33) | 2 (50) | 16 (22) | 1 (100) |
| SVR  GT 1a  GT 1b  GT 3  GT 4  GT 5  GT 6 | 140 (68)  37/66 (56)  58/87 (67)  23/24 (96)  11/12 (92)  1/1 (100)  2/3 (67) | 30 (60)  11/24 (46)  17/23 (74)  n/a  n/a  n/a  n/a | 52 (67)  15/27 (56)  32/44 (73)  n/a  n/a  n/a  n/a | 2 (50)  0/1 (0)  2/3 (67)  n/a  n/a  n/a  n/a | 56 (78)  11/14 (79)  7/17 (41)  23/24 (96)  11/12 (92)  1/1 (100)  2/3 (67) | 0 (0)  n/a  n/a  n/a  n/a  n/a  n/a |

Table legend: SVR rates were calculated taking all patients with a documented 12-week FU after EoT into account. Values shown are percentages and counts and mean values with the corresponding standard deviation. Abbreviations: EoT, end of treatment; FU, follow-up; DAA, direct acting antiviral; GT, genotype; HCV, hepatitis C virus; HIV, human immunodeficiency virus; IFN, interferon; SD, standard deviation.
