# Supplementary material for: Treatment and re-treatment results of HCV patients in the DAA era
Source: PLoS One. 2020 May 5;15(5):e0232773. doi: 10.1371/journal.pone.0232773 (PMC7200014; doi:10.1371/journal.pone.0232773)
Supplement: S3 Table — Baseline characteristics of each special cohort are depicted in the upper part of the table, the lower part of the table shows the outcome of treatment regimens. Next to each treatment regimen in the lower part of the table, the absolute and relative number of patients treated with each regimen is shown, followed by the respective SVR rate (read: n (%) / SVR%). SVR rates were calculated only in patients with a documented 12-week FU after EoT. Values shown are percentages and counts and mean or median values with the corresponding standard deviation or IQR. Abbreviations: BOC, boceprevir; DAA, direct-acting antiviral; DCV, daclatasvir; DSV, dasabuvir; EBR, elbasvir; EoT, end of treatment; FU, follow-up; GLE, glecaprevir; GZR, grazoprevir; HCV, hepatitis C virus; HIV, human immunodeficiency virus; IFN, interferon; IQR, interquartile range; LDV, ledipasvir; MELD, Model for End-Stage Liver Disease; n/a, not applicable; OBV, ombitasvir; PIB, pibrentasvir; PTV, paritaprevir; r, ritonavir; RBV, ribavirin; SD, standard deviation; SIM, simeprevir; SOF, sofosbuvir; SVR, sustained virological response; TVR, telaprevir; VEL, velpatasvir; VOX, voxilaprevir. (DOCX) [file pone.0232773.s003.docx]

**S3 Table. Baseline characteristics and treatment results in special cohorts**

| **Special cohorts**  **No of therapies** | **Liver transplanted patients, n=63 (%)** | **Liver cirrhosis**  **n=320 (%)** | **People with HIV**  **n=49 (%)** | **Liver cirrhosis and HCC, n=24 (%)** |
| --- | --- | --- | --- | --- |
| Age (y, mean ± SD) | 58.2 ± 9.5 | 56.4 ± 10.9 | 50.2 ± 9.0 | 61.5 ± 6.8 |
| Sex (male/female) | 37 (59) / 26 (41) | 205 (64) / 115 (36) | 37 (76) / 12 (24) | 20 (83) / 4 (17) |
| Liver transplanted patients | 63 (100) | 12 (4) | 0 (0) | 0 (0) |
| People with HIV | 0 (0) | 11 (3) | 49 (100) | 0 (0) |
| Liver cirrhosis  Child-Pugh score A/B/C  Median MELD score (IQR) | 12 (19)  3/9/0  7.0 (6.3, 7.8) | 320 (100)  238/79/3  8.0 (7.0, 10.5) | 11 (22)  7/3/1  10 (8.5, 12.0) | 24 (100)  15/9/0  9.5 (8.0, 12.0) |
| IFN-experienced | 46 (73) | 137 (43) | 23 (47) | 9 (38) |
| HCV Genotype  1 (a/b/c/unclassified)  2  3  4  5  6  2k/1b | 46 [73] (16/26/2/2)  5 (8)  7 (11)  5 (8)  0 (0)  0 (0)  0 (0) | 236 [74] (107/119/0/10)  7 (2)  53 (17)  20 (6)  1 (0)  2 (0)  1 (0) | 25 [51] (19/6/0/0)  0 (0)  16 (33)  6 (12)  1 (2)  1 (2)  0 (0) | 16 [67] (9/5/0/2)  1 (4)  5 (21)  2 (8)  0 (0)  0 (0)  0 (0) |
| **Outcome parameters** |  |  |  |  |
| Lost to follow-up | 2 (3) | 24 (8) | 4 (8) | 1 (4) |
| Documented 12-wk FU after EoT | 61 (97) | 296 (92) | 45 (92) | 23 (96) |
| Virological relapse | 8 (13) | 66 (22) | 4 (8) | 8 (35) |
| SVR complete cohort | 53 (87) | 230 (78) | 41 (91) | 15 (65) |
| BOC+IFN [SVR%] | 2 (3) / 50% | 9 (3) / 33% | 2 (4) / 100% | 1 (4) / 100% |
| DCV+IFN [SVR%] | n/a | 1 (0) / 0% | n/a | n/a |
| TVR+IFN [SVR%] | 5 (8) / 40% | 28 (9) / 54% | 4 (9) / 50% | 1 (4) / 0% |
| SOF+IFN [SVR%] | 2 (3) / 0% | 23 (8) / 61% | 4 (9) / 75% | n/a |
| SIM+IFN [SVR%] | n/a | 1 (0) / 0% | n/a | 1 (4) / 0% |
| SOF±RBV [SVR%] | 14 (22) / 86% | 11 (4) / 73% | 4 (9) / 100% | 1 (4) / 0% |
| SOF+DCV [SVR%] | 5 (8) / 100% | 22 (7) / 82% | 5 (11) / 100% | 3 (13) / 33% |
| SOF+SIM [SVR%] | 8 (13) / 100% | 18 (6) / 72% | n/a | 1 (4) / 100% |
| GZR/EBR [SVR%] | 1 (2) / 100% | 8 (3) / 100% | 1 (2) / 100% | n/a |
| GLE/PIB [SVR%] | n/a | 1 (0) / 100% | n/a | n/a |
| OBV/PTV/r [SVR%] | n/a | 2 (1) / 100% | n/a | n/a |
| OBV/PTV/r+DSV [SVR%] | n/a | 20 (7) / 90% | 1 (2) / 100% | 1 (4) / 100% |
| SOF/LDV [SVR%] | 22 (36) / 100% | 111 (38) / 83% | 14 (31) / 93% | 9 (39) / 67% |
| SOF/VEL [SVR%] | 1 (2) / 100% | 33 (11) / 94% | 10 (22) / 100% | 5 (22) / 100% |
| SOF/VEL/VOX [SVR%] | 1 (2) / 100% | 8 (3) / 88% | n/a | n/a |

Table legend: Baseline characteristics of each special cohort are depicted in the upper part of the table; the lower part of the table shows the outcome of treatment regimens. Next to each treatment regimen in the lower part of the table, the absolute and relative number of patients treated with each regimen is shown, followed by the respective SVR rate (read: n (%) / SVR%). SVR rates were calculated only in patients with a documented 12-week FU after EoT. Values shown are percentages and counts and mean or median values with the corresponding standard deviation or IQR.

Abbreviations: BOC, boceprevir; DAA, direct-acting antiviral; DCV, daclatasvir; DSV, dasabuvir; EBR, elbasvir; EoT, end of treatment; FU, follow-up; GLE, glecaprevir; GZR, grazoprevir; HCV, hepatitis C virus; HIV, human immunodeficiency virus; IFN, interferon; IQR, interquartile range; LDV, ledipasvir; MELD, Model for End-Stage Liver Disease; n/a, not applicable; OBV, ombitasvir; PIB, pibrentasvir; PTV, paritaprevir; r, ritonavir; RBV, ribavirin; SD, standard deviation; SIM, simeprevir; SOF, sofosbuvir; SVR, sustained virological response; TVR, telaprevir; VEL, velpatasvir; VOX, voxilaprevir.
