# Supplementary material for: Treatment and re-treatment results of HCV patients in the DAA era
Source: PLoS One. 2020 May 5;15(5):e0232773. doi: 10.1371/journal.pone.0232773 (PMC7200014; doi:10.1371/journal.pone.0232773)
Supplement: S4 Table — Abbreviations: DAA, direct-acting antiviral; DCV, daclatasvir; DSV, dasabuvir; EBR, elbasvir; GLE, glecaprevir; GT, genotype; GZR, grazoprevir; RAS, resistance-associated substitution; LDV, ledipasvir; n/a, not applicable; OBV, ombitasvir; PIB, pibrentasvir; PTV, paritaprevir; r, ritonavir; RBV, ribavirin; SIM, simeprevir; SOF, sofosbuvir; SVR, sustained virological response; VEL, velpatasvir; VOX, voxilaprevir. (DOCX) [file pone.0232773.s004.docx]

**S4 Table.** **RAS and SVR rates of first line or re-treatment regimens**

| **Resistance-associated substitutions (RAS)** | **Before initial DAA treatment** | | | | | | **After first DAA treatment and before re-treatment** | | | | | |
| --- | --- | --- | --- | --- | --- | --- | --- | --- | --- | --- | --- | --- |
|  | GT 1a | GT 1b | GT 2 | GT 2k/1b | GT 3 | GT 5 | GT 1a | GT 1b | GT 2 | GT 3 | GT4 | GT 6 |
| **No of patients tested [n]** | 28 | 2 | 1 | 2 | 33 | 1 | 15 | 9 | 3 | 5 | 3 | 1 |
| **Resistance present [n / %]** | 2 (7%) | 1 (50%) | 0 (0%) | 2 (100%) | 6 (18%) | 0 (0%) | 13 (87%) | 9 (100%) | 1 (33%) | 3 (60%) | 3 (100%) | 0 (0%) |
| **in NS5A gene [n]** | 0 | 0 | 0 | 2 | 6 | 0 | 11 | 8 | 0 | 3 | 3 | 0 |
| Q30 R/H | n/a | n/a | n/a | n/a | n/a | n/a | 8 | 1 | n/a | n/a | n/a | n/a |
| M28 A/T | n/a | n/a | n/a | n/a | n/a | n/a | 2 | n/a | n/a | n/a | n/a | n/a |
| L31 I/L/M/V | n/a | n/a | n/a | 2 | n/a | n/a | 3 | 2 | 1 | n/a | n/a | n/a |
| Y93 N/H/Y | n/a | n/a | n/a | n/a | 2 | n/a | 5 | 8 | n/a | 1 | 1 | n/a |
| P58 S | n/a | n/a | n/a | n/a | n/a | n/a | n/a | 1 | n/a | n/a | n/a | n/a |
| A30 A/V/K/T | n/a | n/a | n/a | n/a | 4 | n/a | n/a | n/a | n/a | 2 | n/a | n/a |
| L28 M/R | n/a | n/a | n/a | n/a | n/a | n/a | n/a | n/a | n/a | n/a | 2 | n/a |
| **in NS5B gene [n]** | 0 | 1 | 0 | 0 | 0 | 0 | 1 | 5 | 1 | 0 | 0 | 0 |
| N444 D | n/a | n/a | n/a | n/a | n/a | n/a | 1 | n/a | n/a | n/a | n/a | n/a |
| M414 V | n/a | n/a | n/a | n/a | n/a | n/a | n/a | 1 | n/a | n/a | n/a | n/a |
| C316 N | n/a | 1 | n/a | n/a | n/a | n/a | n/a | 4 | n/a | n/a | n/a | n/a |
| S556 G | n/a | n/a | n/a | n/a | n/a | n/a | n/a | 4 | n/a | n/a | n/a | n/a |
| L159 F | n/a | n/a | n/a | n/a | n/a | n/a | n/a | 1 | n/a | n/a | n/a | n/a |
| **in NS3 gene [n]** | 2 | 0 | 0 | 0 | 0 | 0 | 3 | 4 | 1 | 0 | 0 | 0 |
| Q80 K | 2 | n/a | n/a | n/a | n/a | n/a | 3 | n/a | n/a | n/a | n/a | n/a |
| Y56 F | n/a | n/a | n/a | n/a | n/a | n/a | n/a | 1 | n/a | n/a | n/a | n/a |
| L36 V | n/a | n/a | n/a | n/a | n/a | n/a | n/a | 1 | n/a | n/a | n/a | n/a |
| I170 V | n/a | n/a | n/a | n/a | n/a | n/a | n/a | 1 | n/a | n/a | n/a | n/a |
| S112 R | n/a | n/a | n/a | n/a | n/a | n/a | n/a | n/a | 1 | n/a | n/a | n/a |
| D168 A/D/E/T | n/a | n/a | n/a | n/a | n/a | n/a | 1 | 2 | n/a | n/a | n/a | n/a |
| **Treatment with [n / SVR%]** |  | | | | | | | | | | | |
| OBV/PTV/r+DSV | 1 (100%) | 1 (100%) | n/a | n/a | n/a | n/a | 1 (100%) | n/a | n/a | n/a | n/a | n/a |
| GZR/EBR | 1 (100%) | 1 (100%) | n/a | n/a | n/a | n/a | n/a | n/a | n/a | n/a | n/a | n/a |
| GLE/PIB | 11 (100%) | n/a | n/a | n/a | 8 (100%) | 1 (100%) | n/a | n/a | n/a | n/a | n/a | n/a |
| SOF/LDV | 10 (100%) | n/a | n/a | n/a | n/a | n/a | n/a | 1 (100%) | n/a | n/a | n/a | n/a |
| SOF/LDV + RBV | n/a | n/a | n/a | n/a | n/a | n/a | n/a | n/a | n/a | n/a | 1 (100%) | n/a |
| SOF+SIM | 1 (100%) | n/a | n/a | n/a | n/a | n/a | 1 (100%) | 2 (100%) | n/a | n/a | 1 (100%) | n/a |
| SOF/VEL | 3 (100%) | n/a | 1 (100%) | 2 (100%) | 25 (96%) | n/a | n/a | n/a | n/a | n/a | n/a | n/a |
| SOF+DAC | n/a | n/a | n/a | n/a | n/a | n/a | 1 (0%) | n/a | n/a | 3 (100%) | n/a | n/a |
| SOF/VEL/VOX | n/a | n/a | n/a | n/a | n/a | n/a | 6 (83%) | 3 (100%) | 2 (100%) | 2 (100%) | 1 (100%) | n/a |

Table legend: Abbreviations: DAA, direct-acting antiviral; DCV, daclatasvir; DSV, dasabuvir; EBR, elbasvir; GLE, glecaprevir; GT, genotype; GZR, grazoprevir; RAS, resistance-associated substitution; LDV, ledipasvir; n/a, not applicable; OBV, ombitasvir; PIB, pibrentasvir; PTV, paritaprevir; r, ritonavir; RBV, ribavirin; SIM, simeprevir; SOF, sofosbuvir; SVR, sustained virological response; VEL, velpatasvir; VOX, voxilaprevir.
